# Supplementary material for: Tumors attenuating the mitochondrial activity in T cells escape from PD-1 blockade therapy
Source: eLife. 2020 Mar 3;9:e52330. doi: 10.7554/eLife.52330 (PMC7105382; doi:10.7554/eLife.52330)
Supplement: Supplementary file 1. [file elife-52330-supp1.docx]

**List of primers for quantifying mouse gene transcripts by qRT-PCR.**

| Transcript | Forward primer | Reverse primer |
| --- | --- | --- |
| CD39 | TACCACCCCATCTGGTCATT | GGACGTTTTGTTTGGTTGGT |
| CD73 | CAAATCCCACACAACCACTG | TGCTCACTTGGTCACAGGAC |
| COX2 | CAAGGGAGTCTGGAACATTG | ACCCAGGTCCTCGCTTATGA |
| mPGES1 | ATGAGTACACGAAGCCGAGG | CCAGTATTACAGGAGTGACCCAG |
| IDO1 | CACTGAGCACGGACGGACTGAGA | TCCAATGCTTTCAGGTCTTGACGC |
| ß-actin | TATTGGCAACGAGCGGTTCC | GGCATAGAGGTCTTTACGGATGT |
| PGC-1α | CGGAAATCATATCCAACCAG | TGAGGACCGCTAGCAAGTTTG |
| PGC-1β | GGTGTTCGGTGAGATTGTAGAG | GTGATAAAACCGTGCTTCTGG |
